# Supplementary material for: Modeling glioblastoma heterogeneity as a dynamic network of cell states
Source: Mol Syst Biol. 2021 Sep 16;17(9):e10105. doi: 10.15252/msb.202010105 (PMC8444284; doi:10.15252/msb.202010105)
Supplement: Supplementary file 6 — Source Data for Figure 5 [file MSB-17-e10105-s004.zip › Figure5A_sourcedata/GSEA_3071/hallmarks_stateV.GseaPreranked.1623742546329/HALLMARK_APICAL_JUNCTION.html]

Details for gene set HALLMARK\_APICAL\_JUNCTION[GSEA]

|  || Dataset | state43071 |
| Phenotype | NoPhenotypeAvailable |
| Upregulated in class | na\_neg |
| GeneSet | HALLMARK\_APICAL\_JUNCTION |
| Enrichment Score (ES) | -0.41661075 |
| Normalized Enrichment Score (NES) | -2.2750235 |
| Nominal p-value | 0.0 |
| FDR q-value | 0.0 |
| FWER p-Value | 0.0 |
Table: GSEA Results Summary

  

Fig 1: Enrichment plot: HALLMARK\_APICAL\_JUNCTION      
 Profile of the Running ES Score & Positions of GeneSet Members on the Rank Ordered List

  

| PROBE | GENE SYMBOL | GENE\_TITLE | RANK IN GENE LIST | RANK METRIC SCORE | RUNNING ES | CORE ENRICHMENT || 1 | LIMA1 |  |  | 264 | 1.098 | -0.0337 | No |
| 2 | GAMT |  |  | 327 | 1.040 | -0.0296 | No |
| 3 | SIRPA |  |  | 352 | 1.014 | -0.0185 | No |
| 4 | CDH4 |  |  | 474 | 0.888 | -0.0280 | No |
| 5 | EVL |  |  | 623 | 0.787 | -0.0443 | No |
| 6 | NRXN2 |  |  | 635 | 0.781 | -0.0343 | No |
| 7 | JAM3 |  |  | 704 | 0.745 | -0.0359 | No |
| 8 | ADAM23 |  |  | 758 | 0.719 | -0.0350 | No |
| 9 | TUBG1 |  |  | 776 | 0.712 | -0.0272 | No |
| 10 | CADM3 |  |  | 817 | 0.693 | -0.0242 | No |
| 11 | GNAI2 |  |  | 845 | 0.681 | -0.0189 | No |
| 12 | PKD1 |  |  | 885 | 0.666 | -0.0161 | No |
| 13 | ARHGEF6 |  |  | 935 | 0.647 | -0.0155 | No |
| 14 | LAMB3 |  |  | 939 | 0.646 | -0.0061 | No |
| 15 | MDK |  |  | 950 | 0.640 | 0.0019 | No |
| 16 | KCNH2 |  |  | 955 | 0.638 | 0.0110 | No |
| 17 | MYH10 |  |  | 1172 | 0.571 | -0.0217 | No |
| 18 | PARD6G |  |  | 1174 | 0.571 | -0.0131 | No |
| 19 | HADH |  |  | 1242 | 0.548 | -0.0175 | No |
| 20 | PIK3R3 |  |  | 1327 | 0.526 | -0.0255 | No |
| 21 | YWHAH |  |  | 1407 | 0.502 | -0.0329 | No |
| 22 | SHROOM2 |  |  | 1431 | 0.497 | -0.0296 | No |
| 23 | DLG1 |  |  | 1491 | 0.482 | -0.0335 | No |
| 24 | VAV2 |  |  | 1528 | 0.475 | -0.0331 | No |
| 25 | NLGN3 |  |  | 2078 | 0.376 | -0.1327 | No |
| 26 | CDH6 |  |  | 2101 | 0.374 | -0.1312 | No |
| 27 | ADAM9 |  |  | 2228 | 0.359 | -0.1498 | No |
| 28 | AKT3 |  |  | 2292 | 0.351 | -0.1565 | No |
| 29 | NLGN2 |  |  | 2346 | 0.342 | -0.1614 | No |
| 30 | SORBS3 |  |  | 2361 | 0.341 | -0.1588 | No |
| 31 | CRAT |  |  | 2458 | 0.327 | -0.1722 | No |
| 32 | TAOK2 |  |  | 2487 | 0.324 | -0.1726 | No |
| 33 | COL9A1 |  |  | 2756 | 0.294 | -0.2195 | No |
| 34 | PTK2 |  |  | 2794 | 0.290 | -0.2221 | No |
| 35 | PIK3CB |  |  | 2890 | 0.281 | -0.2360 | No |
| 36 | AKT2 |  |  | 3094 | 0.260 | -0.2710 | No |
| 37 | NECTIN1 |  |  | 3141 | 0.256 | -0.2759 | No |
| 38 | MMP2 |  |  | 3234 | -0.257 | -0.2896 | No |
| 39 | ITGB1 |  |  | 3614 | -0.317 | -0.3575 | No |
| 40 | NEGR1 |  |  | 3631 | -0.320 | -0.3556 | No |
| 41 | MAP3K20 |  |  | 3668 | -0.326 | -0.3575 | No |
| 42 | BAIAP2 |  |  | 3844 | -0.357 | -0.3856 | No |
| 43 | NF1 |  |  | 3882 | -0.364 | -0.3871 | No |
| 44 | AMIGO2 |  |  | 3900 | -0.367 | -0.3847 | No |
| 45 | EPB41L2 |  |  | 3911 | -0.369 | -0.3809 | No |
| 46 | TSPAN4 |  |  | 3916 | -0.369 | -0.3759 | No |
| 47 | RASA1 |  |  | 3944 | -0.377 | -0.3753 | No |
| 48 | IRS1 |  |  | 3971 | -0.383 | -0.3744 | No |
| 49 | ZYX |  |  | 4029 | -0.395 | -0.3792 | No |
| 50 | INPPL1 |  |  | 4121 | -0.415 | -0.3903 | No |
| 51 | ARPC2 |  |  | 4259 | -0.458 | -0.4095 | Yes |
| 52 | NECTIN2 |  |  | 4260 | -0.458 | -0.4024 | Yes |
| 53 | MYL12B |  |  | 4267 | -0.462 | -0.3965 | Yes |
| 54 | CD99 |  |  | 4339 | -0.484 | -0.4026 | Yes |
| 55 | PARVA |  |  | 4352 | -0.487 | -0.3974 | Yes |
| 56 | SKAP2 |  |  | 4381 | -0.495 | -0.3951 | Yes |
| 57 | TJP1 |  |  | 4489 | -0.536 | -0.4074 | Yes |
| 58 | VCAN |  |  | 4491 | -0.537 | -0.3993 | Yes |
| 59 | MPZL1 |  |  | 4500 | -0.541 | -0.3925 | Yes |
| 60 | ADAMTS5 |  |  | 4528 | -0.554 | -0.3891 | Yes |
| 61 | BMP1 |  |  | 4559 | -0.569 | -0.3861 | Yes |
| 62 | MVD |  |  | 4604 | -0.591 | -0.3854 | Yes |
| 63 | MSN |  |  | 4698 | -0.644 | -0.3933 | Yes |
| 64 | RRAS |  |  | 4717 | -0.657 | -0.3866 | Yes |
| 65 | RSU1 |  |  | 4738 | -0.670 | -0.3801 | Yes |
| 66 | CD274 |  |  | 4743 | -0.673 | -0.3705 | Yes |
| 67 | ACTN1 |  |  | 4775 | -0.695 | -0.3657 | Yes |
| 68 | CNN2 |  |  | 4840 | -0.749 | -0.3664 | Yes |
| 69 | FLNC |  |  | 4856 | -0.759 | -0.3575 | Yes |
| 70 | CALB2 |  |  | 4858 | -0.762 | -0.3460 | Yes |
| 71 | NF2 |  |  | 4864 | -0.769 | -0.3350 | Yes |
| 72 | THY1 |  |  | 4874 | -0.781 | -0.3247 | Yes |
| 73 | VASP |  |  | 4892 | -0.802 | -0.3156 | Yes |
| 74 | CAP1 |  |  | 5002 | -0.934 | -0.3221 | Yes |
| 75 | ITGA10 |  |  | 5016 | -0.960 | -0.3097 | Yes |
| 76 | ACTG1 |  |  | 5017 | -0.961 | -0.2949 | Yes |
| 77 | MYH9 |  |  | 5090 | -1.125 | -0.2913 | Yes |
| 78 | SHC1 |  |  | 5094 | -1.133 | -0.2744 | Yes |
| 79 | NEXN |  |  | 5133 | -1.251 | -0.2624 | Yes |
| 80 | TGFBI |  |  | 5136 | -1.259 | -0.2433 | Yes |
| 81 | ITGA2 |  |  | 5201 | -1.557 | -0.2316 | Yes |
| 82 | ITGA3 |  |  | 5204 | -1.561 | -0.2078 | Yes |
| 83 | VCL |  |  | 5222 | -1.678 | -0.1852 | Yes |
| 84 | LAYN |  |  | 5227 | -1.727 | -0.1593 | Yes |
| 85 | EGFR |  |  | 5230 | -1.765 | -0.1324 | Yes |
| 86 | CDH11 |  |  | 5245 | -1.943 | -0.1050 | Yes |
| 87 | FBN1 |  |  | 5263 | -2.234 | -0.0738 | Yes |
| 88 | ACTG2 |  |  | 5276 | -2.506 | -0.0374 | Yes |
| 89 | MYL9 |  |  | 5280 | -2.643 | 0.0029 | Yes |
Table: GSEA details [plain text format]

  

Fig 2: HALLMARK\_APICAL\_JUNCTION: Random ES distribution      
 Gene set null distribution of ES for **HALLMARK\_APICAL\_JUNCTION**
